# Antibacterial Potency of an Active Compound from *Sansevieria trifasciata* Prain: An Integrated In Vitro and In Silico Study

**DOI:** 10.3390/molecules28166096

**Published:** 2023-08-17

**Authors:** Henny Kasmawati, Ruslin Ruslin, Arfan Arfan, Nurramadhani A. Sida, Dimas Isnu Saputra, Eli Halimah, Resmi Mustarichie

**Affiliations:** 1 Department of Pharmacy, Faculty of Pharmacy, Universitas Halu Oleo, Kendari 93232, Indonesia; mahaleo241@yahoo.co.id (R.R.); arfan09@uho.ac.id (A.A.); apt.nurramadhani08@uho.ac.id (N.A.S.); dimasisnusaputra@gmail.com (D.I.S.); 2 Department of Pharmacology and Clinical Pharmacy, Faculty of Pharmacy, Universitas Padjadjaran, Bandung 45363, Indonesia; eli.halimah@unpad.ac.id; 3 Department of Analytical Pharmacy and Medicinal Chemistry, Faculty of Pharmacy, Universitas Padjadjaran, Bandung 45363, Indonesia

**Keywords:** antibacterial, molecular dynamics, *Sansevieria trifasciata* Prain, 5-methyl-11-(2-oxopyridin-1(2H)-yl)undecaneperoxoicacid, β-ketoacyl-ACP synthase, TyrRS

## Abstract

*Sansevieria trifasciata* Prain holds great potential as a valuable asset in pharmaceutical development. In this study, our focus is to explore and assess the antibacterial activity of various components derived from this plant, including extracts, fractions, subfractions, and isolates, explicitly targeting two common bacteria: *Escherichia coli* and *Streptococcus aureus*. The isolated compound, identified as a derivative pyridone alkaloid (5-methyl-11-(2-oxopyridin-1(2H)-yl)undecaneperoxoicacid), demonstrates notable antibacterial effects. The extracts, fractions, subfractions, and isolates reveal significant bacterial growth reductions (*p* < 0.05). The minimum inhibitory concentration (MIC) values for *Escherichia coli* were 1.95 ppm, 3.9 ppm, 15.62 ppm, and 7.81 ppm, respectively, while the MIC values for *Streptococcus aureus* were 1.95 ppm, 1.95 ppm, 15.62 ppm, and 7.81 ppm, respectively. Computational analysis showed the isolates’ interaction with key residues on the active site of β-ketoacyl-ACP synthase from *Escherichia coli* and TyrRS from *Streptococcus aureus*. The findings indicate that the isolates exhibit a strong affinity for specific residues, including His333, Cys163, and Phe392 in β-ketoacyl-ACP synthase, as well as Arg88, His117, Glu160, and Gln213 in TyrRS. Comparative energy calculations using MMPBSA demonstrate the isolates’ favorable binding energy (−104,101 kJ/mol for β-ketoacyl-ACP synthase and −81,060 kJ/mol for TyrRS) compared to ciprofloxacin. The elucidated antibacterial activity and molecular interactions of the isolates present valuable knowledge for future in vitro studies, facilitating the development of novel antibacterial agents targeting diverse bacterial strains.

## 1. Introduction

*Sansevieria trifasciata* Prain is a potential plant to be developed as a medical resource. This plant has been traditionally used since ancient times [1]. Therefore, numerous studies have been conducted to prove its pharmacological effects and determine the chemical constituents of these plants [2,3]. Phytochemical studies showed the presence of trifasciatine C dihydrochalcone derivatives [4], steroidal saponins such as trifasciatoside A-I [5], trifasciatosides K-N, 1,2-(dipalmitoyl)-3-O-β-D-galactopyranosylglycerol, aconitic acid, 1-methyl aconitic acid [4], Neoruscogenin, ruscogenin, sansevierigenin, Luvigenin [6], homoisflavonoid trifasciatin A-B [7], pregnane glucoside [8], alkaloid 1-Acetyl-β-carboline, methyl pyrophaeophorbide A, and oliveramine, and flavonoids such as (2S)-3′, 4′-methylenedioxy-5, 7-dimethoxyflavane, monoterpenes digiprolactone, phenolic methyl gallate, and fatty acid trichosanic acid [3].

*S. trifasciata* Prain is primarily recognized for its ornamental value [9]. However, researchers believe that this plant’s chemical composition can potentially combat various diseases, including its potential as an antibacterial agent [10]. *S. trifasciata* Prain ethyl acetate extract can inhibit the growth of *Escherichia coli* and *Streptococcus aureus* bacteria [11], ethanol extract and fraction can inhibit the proliferation of *Pseudomonas aeruginosa*, and it is suspected that the Neophytadiene compound identified by GC-MS is one of the compounds that play a role in this activity [1]. Butanol extract can inhibit the growth of *Salmonella* spp. and *Streptococcus aureus* bacteria [12].

Expanding on our prior research trials, we have employed LC-MS/MS methods to detect bioactive compounds derived from *S. trifasciata* Prain, explicitly highlighting its anti-alopecia properties. These significant findings have ignited our interest in delving deeper into this plant’s extensive range of pharmacological activities [3]. There have been numerous publications regarding the antibacterial activity of *S. trifasciata* Prain, but antibacterial studies of isolated compounds and their underlying molecular mechanisms have yet to be reported. In this study, our present focus is on investigating the antibacterial properties of *S. trifasciata* Prain. Therefore, this study aimed to isolate bioactive compounds from the plant using the bioassay-guided method and comprehensively evaluate their antibacterial properties [13]. This encompassed thorough assessments of extracts, fractions, subfractions, and isolates using in vitro and in silico methods, ensuring a broad evaluation of their antibacterial properties against *Escherichia coli* and *Streptococcus aureus* bacteria [14].

The bacteria *Escherichia coli* and *Streptococcus aureus* have several crucial enzymes that can serve as drug targets, particularly β-ketoacyl-ACP synthase from *Escherichia coli* and TyrRS from *Streptococcus aureus*. β-ketoacyl-acyl carrier protein (ACP) synthases are the key regulators of fatty acid synthesis in *Escherichia coli* [15,16]. β-ketoacyl-ACP synthase is involved in condensation reactions during fatty acid biosynthesis [16]. In addition, tyrosyl-tRNA synthetase (TyrRS) is an enzyme that plays a crucial role in protein synthesis by attaching the amino acid tyrosine to its corresponding tRNA molecule [17,18]. In *Staphylococcus aureus*, TyrRS has been found to have additional functions beyond its canonical role in protein synthesis and was shown to interact with the bacterial cell wall and contribute to its maintenance and integrity [18]. This comprehensive approach has provided a comprehensive understanding of the antibacterial activity exhibited by *S. trifasciata* Prain, enabling us to uncover its therapeutic potential against bacterial infections.

## 2. Results

### 2.1. Maceration and Isolation

**Figure 1:**
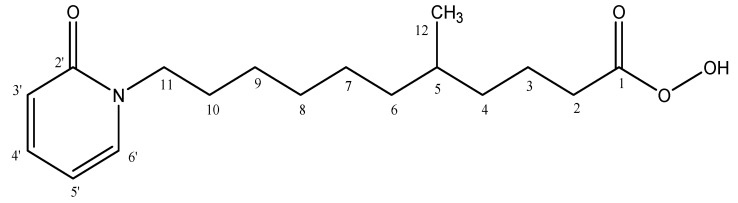
The structure of 5-methyl-11-(2-oxopyridin-1(2H)-yl)undecaneperoxoicacid compound. The structure consists of: C1 = C quarternary of carbonyl ester in peroxoic acid structure; C2, C3, C4 = methylene (CH_2_); C5 = C quarternary; C6, C7, C8, C9, C10, and C11 = methylene (CH_2_); C2’= C quarternary; C3’, C4’, C5, and C6’ = methyne (CH). C12 = methyl (CH_3_) in the aromatic ring of the oxopyridin structure.

Simplicia powder from the leaves of *S. trifasciata* Prain was macerated with ethanol to obtain a viscous ethanol extract (450 g) with a 13.35% (*w*/*w*) yield after the extract was evaporated. Separation of the extract by trituration produced filtrate as the n-hexane fraction and residue, which was triturated again using ethyl acetate solvent. The sample was separated with n-hexane solvent to remove the ballast substance from the sample. Trituration of the residue resulted in an ethyl acetate fraction (147.6 g) with a 36.9% (*w*/*w*) yield. Further separation of the ethyl acetate fraction produced alkaloid derivatives. The structure of this compound was characterized using ^1^H-NMR, ^13^C-NMR, distortionless enhancement by polarization transfer (DEPT), heteronuclear multiple quantum coherence (HMQC), heteronuclear multiple bond correlation (HMBC), and ^1^H-^1^H correlated spectroscopy (COSY) were used to obtain 5-methyl-11-(2-oxopyridine-1(2H)-yl)undecaneperoxoicacid (Figure 1). HMBC spectroscopy is used to ascertain the connectivity between ^1^H and ^13^C over extended distances, 2–4 bonds from the proton [19], whereas HMQC is used for protons and carbon with a distance of 1 bond. DEPT can differentiate among signals from methyl (-CH_3_), methylene (-CH_2_), methyne (-CH), and quaternary (-Cq) carbon [20], whereas COSY is used to determine the correlations among protons within a single carbon atom as well as among protons in adjacent carbon atoms [21].

### 2.2. Characterization of 5-Methyl-11-(2-oxopyridin-1(2H)-yl)undecaneperoxoicacid Compound

The results of the C-NMR spectral characterization show the presence of 17 carbon atoms with different types of groups based on their chemical shifts. The ^13^C-NMR results show that the carbons at chemical shifts 173.2 and 162.08 are a carbonyl group, which is an acid derivative (C=O); those on chemical shifts 132.069, 126.972, 125.955, and 115.646 are an aromatic carbon group (C-H); and those on chemical shifts 33.731, 31.83, 29.872, 29.546, 29.44, 29.277, 29.248, 29.085, 24.458, 22.635, and 14.495 is an alkyl group (C-H, C-H_2_, and C-H_3_-). The DEPT-135 data at chemical shifts 173.2 and 162.08 show a type of quaternary carbon (C), those at chemical shifts 132.069, 126.972, 125.955, 115.646, and 29.872 indicate a type of methine carbon (C-H), and those at chemical shifts 33.731, 31.83, 29.546, 29.44, 29.277, 29.248, 29.085, 24.458, and 22.635 indicate a type of methylene carbon (CH_2_) (Figure S1).

The ^1^H-NMR data show 27 protons with 10 signal peaks in the ^1^H-NMR spectrum. There are signals of aromatic proton groups (Ar-H) at chemical shifts 7.74 and 6.94 ppm, which come out as doublets with constant coupling (J) = 8, indicating the presence of aromatics with H-H coupling in the ortho position. The HMQC spectra show signals of δc- δH C-3′ (132.069 ppm) with H (7.74 ppm), C-4′ (15.646 ppm) with H (6.77 ppm), C-5′ (126.972 ppm) with H (6.94 ppm), and C-6′ (125.955 ppm) with H (7.03 ppm) (Figure S4). The presence of a signal on the HNMR δH 7.03 (6.77 ppm) and HMBC signals at C-5’ and C-6’ (Figure S5) indicates that the signal is part of an aromatic ring. The results of the ^1^H-^1^H of COSY support the aromatic HNMR, that is, the presence of a signal on the ^13^C NMR spectrum, at δH 7.74–6.77, 7.03–6.94, and 6.94–6.77 (Figure S6). This strengthens the suspicion that the isolated compound's structure contains an aromatic ring. Atom C-2’ in the aromatic ring is predicted as carbonyl (C=O). The chemical shift of carbon C-2’ appears in the deshielding area (δC 162.084 ppm), indicating electron-withdrawing group oxygen. Additionally, based on the data from the DEPT, atom C-2’ is a C quarter and is correlated to the hydrogen elements on C-3’ and C-4’ based on the HMBC data. The hydroxyl group (R-O-H) was detected as a singlet ^1^H at δH 3.94 ppm, and an ester carbon was detected at δC 173.2 ppm. The prediction of the presence of an ester carbon atom is supported by the DEPT data, indicating that the carbon atom at δC 173.2 is a C quarter and appears in the deshielding region. According to the HMBC spectra, this ester carbon atom correlates with the H atoms on C-2 and C-3, whereas the DEPT data suggest that C-2 and C-3 are methylene (CH_2_). This indicates that the ester carbon's companion is a carbon chain, while the atom O from esters has a hydroxyl group as a substituent. This is explained by the HMBC results, which reveal a correlation between the H atom of the hydroxy group and the C ester atom. Based on these data, the compound is suspected to have a peroxidic acid group. The presence of a methyl substituent on the C-5 atom was predicted based on the DEPT and HMBC data. The DEPT data show that the type of C-5 is methine (CH), and the atom C-12 is indicated as methyl (CH_3_). C-5 is related to the H atom at δH 0.79 (CH_3_) in HMBC. Therefore, it is considered that the C-5 atom has a -CH_3_ substituent.

### 2.3. Computational Study

#### 2.3.1. Molecular Docking

**Figure 2:**
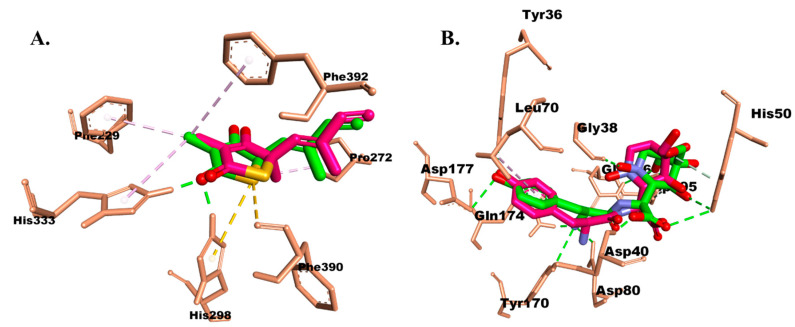
Superimposed image of the interaction between the co-crystallized native ligand (green) and the docked pose (pink) of (**A**) TLM in β-ketoacyl-ACP synthase from *Escherichia coli* and (**B**) 629 in TyrRS from *Streptococcus aureus*.

The preferred binding modes of a ligand to an enzyme were predicted through molecular docking analyses, as evidenced by binding affinity and residual amino acid interactions. Additionally, we focused on identifying the key amino acid residues in the essential enzymes of *Escherichia coli* and *Streptococcus aureus*, which are β-ketoacyl-acyl carrier protein synthase (β-ketoacyl-ACP synthase) and tyrosyl-tRNA synthetase (TyrRS), respectively. These residues play a crucial role in stabilizing the bond between the ligand and the enzyme. In this simulation, interaction predictions were performed with the two target enzymes. To assess the methodology, the co-crystal ligand was redocked to measure its RMSD. The optimal conformation of the native ligand (TLM and 629) is shown in Figure 2. A lower RMSD value indicates a better ability of the docking parameters to mimic the native ligand’s conformation, as observed in X-ray crystallography. TLM, in complex with β-ketoacyl-ACP synthase, and 629, in complex with TyrRS, were chosen as the validation parameters. These compounds exhibit inhibitory effects on their target proteins and provide crucial insights into the active binding sites of these targets. Thus, they served as the reference coordinates for simulating the interactions between the isolated compounds and the target proteins [22].

The β-ketoacyl-ACP synthase from *Escherichia coli* in complex with TLM was chosen as the protein model based on the known characteristics of the ligand as an ACP synthase inhibitor. Redocking analysis of the co-crystallized TLM to the binding site of *Escherichia coli* ACP synthase revealed a similar pose to the reported X-ray crystallography (RMSD 0.685 Å) (Figure 2A), where hydrogen bonds (H-bonds) were observed between the carbonyl group of TLM and the residues His298 and His333. Meanwhile, the drug candidate 629 complexed with TyrRS from *Streptococcus aureus* successfully mimicked the X-ray crystallography pose with an RMSD of 1.437 Å (Figure 2B).

In this study, the native ligands exhibited stronger affinities compared to ciprofloxacin and isolate towards the enzymes found in *Escherichia coli* and *Streptococcus aureus*, with energies of −7.96 kcal/mol for TLM and −9.35 kcal/mol for 629. For the target enzyme β-ketoacyl-ACP synthase from *Escherichia coli*, the isolated compound (−6.72 kcal/mol) showed a nearly equivalent affinity to ciprofloxacin (−6.81 kcal/mol). On the other hand, the isolate displayed a weaker affinity towards the target enzyme TyrRS from *Streptococcus aureus*, with a binding energy of −6.15 kcal/mol compared to ciprofloxacin’s −6.69 kcal/mol (Table 1).

**Figure 3:**
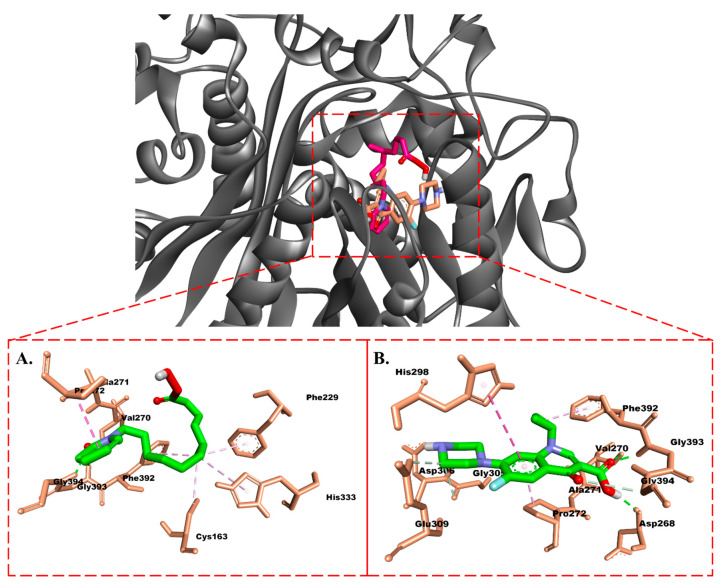
Molecular interactions of (**A**) isolate and (**B**) ciprofloxacin with β-ketoacyl-ACP synthase from *Escherichia coli.* Carbon, oxygen, and nitrogen atoms are marked in green, red, and blue. Amino acid residues are highlighted with a light brown color. The green, gray, and pink dotted lines represent hydrogen bonding, carbon-hydrogen bonds, and hydrophobic interactions, respectively.

Hydrophobic interactions were observed among the methyl groups of TLM and Phe229, Pro272, and Phe392 (Figure 2A). Ciprofloxacin showed four H-bonds with the amino acids Asp268, Val270, Asp306, and Gly393. Hydrophobic interactions of this compound were observed with Ala271, Pro272, His298, and Phe392 (Figure 3B). Interestingly, the carbonyl group of the isolate exhibited different hydrogen bonding interactions compared to TLM, specifically with Gly393 and Gly394. Meanwhile, its hydrophobic interactions resembled those of TLM and ciprofloxacin, but differed in residues Cys163 and Ala271 (Figure 3A).

**Figure 4:**
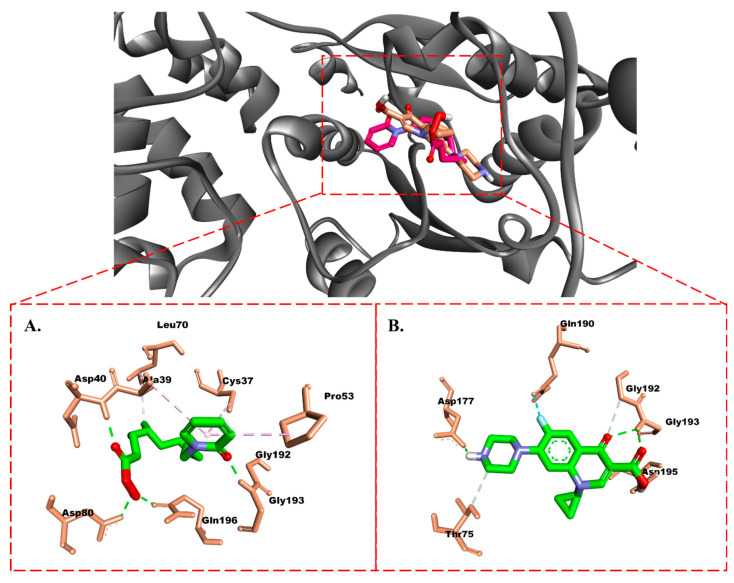
Molecular interactions of (**A**) isolate and (**B**) ciprofloxacin with TyrRS from *Streptococcus aureus.* Carbon, oxygen, and nitrogen atoms are marked in green, red, and blue. Amino acid residues are highlighted with a light brown color. The green, gray, and pink dotted lines represent hydrogen bonding, carbon-hydrogen bonds, and hydrophobic interactions, respectively.

Compound 629 formed hydrogen bonds with the residues Tyr36, Gly38, Asp40, His50, Asp80, Lys84, Tyr170, Gln174, Asp177, and Gly193. These hydrogen bonds were correlated with a higher affinity compared to the other compounds. Additionally, the benzene moiety contributed to hydrophobic interactions with Leu70, which were also observed in the isolated compound. Ciprofloxacin and the isolate formed hydrogen bonds similar to candidate 629, except for the residue Asp195, which interacted with the hydroxyl group of ciprofloxacin, and the residue Gln196, which interacted with the hydroxyl group of the isolate (Figure 4). Interestingly, no hydrophobic interactions were observed for ciprofloxacin, but its fluorine atom interacted with the residue Gln190 (Figure 4B). On the other hand, the benzene ring of the isolated compound also exhibited hydrophobic interactions with the residues Cys37, Ala39, and Pro53 (Figure 4A).

#### 2.3.2. Molecular Dynamics Simulation

##### Root Mean Square Deviation and Fluctuation (RMSD and RMSF)

**Figure 5:**
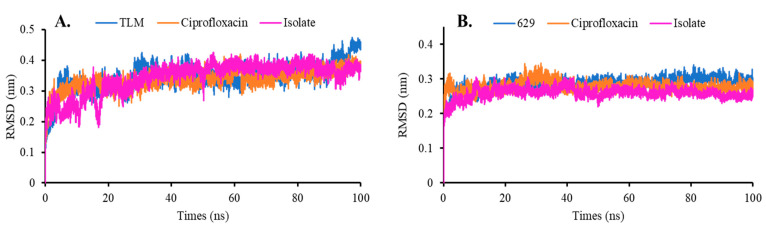
RMSD analysis on complex systems in (**A**) beta-ketoacyl-ACP synthase from *Escherichia coli* and (**B**) TyrRS from *Streptococcus aureus*.

The RMSD values of β-ketoacyl-ACP synthase and TyrRS, as well as the isolate from *S. trifasciata*, compared to ciprofloxacin, can be seen in Figure 5. In a system simulation, the RMSD compares the folded protein structure to the partially or entirely unfolded structure. The RMSD represents the dynamic changes in the protein during the simulation and is crucial for assessing protein stability [23]. During the simulation, both the native ligand and ciprofloxacin were observed to be stable, with average RMSD values of 0.347 nm and 0.337 nm for beta-ketoacyl-ACP synthase, and 0.285 nm and 0.277 nm for TyrRS. Interestingly, the isolate exhibited similar stability to both compounds. In the complex of beta-ketoacyl-ACP synthase from *Escherichia coli*, the isolated compound had an average RMSD of 0.343 nm (Figure 5A), while in TyrRS from *Streptococcus aureus*, the isolated compound had an average RMSD of 0.258 nm (Figure 5B). These findings suggest that the isolated compound can stabilize both target enzymes from *Escherichia coli* and *Streptococcus aureus*, as evidenced by the relatively constant RMSD oscillation during the simulation.

**Figure 6:**
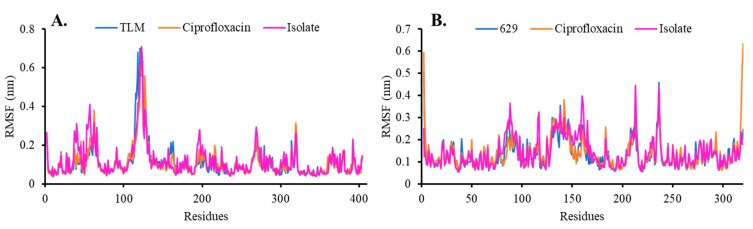
RMSF analysis of complex systems in (**A**) beta-ketoacyl-ACP synthase from Escherichia coli and (**B**) TyrRS from Streptococcus aureus.

The RMSF in the β-ketoacyl-ACP synthase and TyrRS complexes displayed a range of intensity values (Figure 6). The RMSF characterizes the fluctuation of the central carbon atoms of each residue’s coordinates near its reference point during the dynamic simulation, indicating the oscillation of the protein structure [24]. The RMSF profile of β-ketoacyl-ACP synthase did not show significant amino acid fluctuations when complexed with the native ligand (TLM) or ciprofloxacin. High-intensity oscillations were observed at residue Pro123 around 0.71 nm, with moderate oscillations at residues Glu196, Met269, and Asp319 ranging from 0.27 nm to 0.31 nm. Interestingly, in the isolate complex, there were residues with relatively high fluctuations, namely Lys40 and Thr57, with RMSF values of 0.31 nm and 0.41 nm, respectively (Figure 6A). On the other hand, the RMSF profile of TyrRS fluctuated within the range of residues 80–240. Interestingly, the native ligand (629) and ciprofloxacin showed similar residue fluctuations. Residues with relatively high fluctuations included Thr2 and Ser320, with RMSF values of 0.59 nm and 0.63 nm, respectively, located at the carboxyl and amino terminus of the *Streptococcus aureus* bacterial TyrRS (Figure 6B). In addition, residues Lys142, Arg183, and Glu236 also exhibited fluctuations with intensities of 0.38 nm, 0.25 nm, and 0.45 nm, respectively. When complexed with the isolate, four residues showed relatively high fluctuations, namely Arg88, His117, Glu160, and Gln213, with RMSF values of 0.36 nm, 0.32 nm, 0.39 nm, and 0.49 nm. These studies revealed that the isolate caused increased fluctuations in several residues during the simulation.

##### Solvent Accessible Surface Area (SASA) and Radius Gyration (Rg)

**Figure 7:**
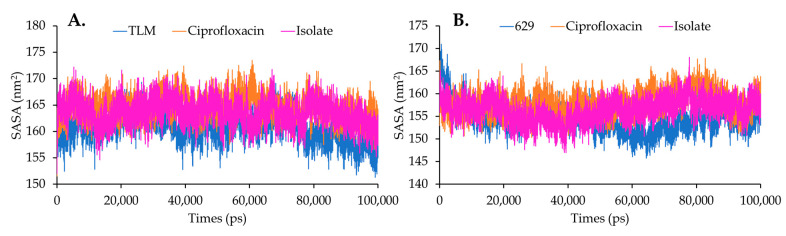
SASA analysis on complex systems in (**A**) beta-ketoacyl-ACP synthase from *Escherichia coli* and (**B**) TyrRS from *Streptococcus aureus*.

The change in the accessible area for solvent is one of the parameters that can describe the folding and stability of a protein complex during a simulation [25]. It can be observed that TLM in the β-ketoacyl-ACP synthase complex decreased in the area accessed by the solvent during the simulation, with an average solvent accessible surface area (SASA) of 160.798 nm^2^ (Figure 7A). Additionally, ciprofloxacin and the isolate exhibited larger but similar areas, with respective SASA values of 164.587 nm^2^ and 163.554 nm^2^. On the other hand, the SASA of TyrRS was smaller when complexed with 629, measuring 154.697 nm^2^, followed by the isolate at 156.446 nm^2^, and ciprofloxacin had the largest SASA at 158.168 nm^2^ (Figure 7B). However, overall, the isolated compound did not significantly affect the changes in SASA for both *Escherichia coli* and *Streptococcus aureus* enzymes.

**Figure 8:**
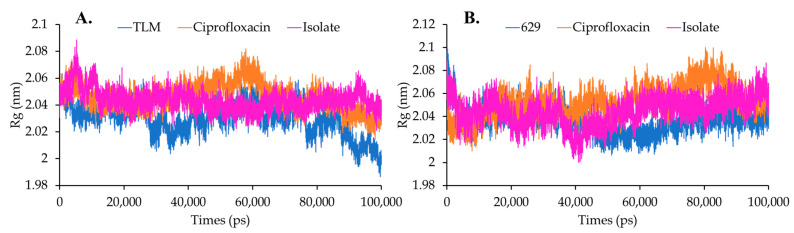
Gyration radius (Rg) analysis on complex systems in (**A**) beta-ketoacyl-ACP synthase from *Escherichia coli* and (**B**) TyrRS from *Streptococcus aureus*.

Protein compactness was assessed during the simulation by measuring the radius of gyration (Rg) of each complex. Protein binding with a ligand affects protein folding and stability, which can be monitored through their pattern and Rg values. Low and consistent Rg values indicate stable protein folding behavior during the simulation [26]. According to the graph (Figure 8A), β-ketoacyl-ACP synthase exhibited a lower Rg value when interacting with TLM, with an average of 2.029 nm.

Interestingly, ciprofloxacin and the isolate demonstrated stable Rg values during the simulation, averaging 2.046 nm and 2.043 nm, respectively. On the other hand, the protein complex of TyrRS with 629 showed a more stable Rg value with an average of 2.037 nm. Furthermore, ciprofloxacin and the isolate complexed with TyrRS exhibited increased compactness, with Rg values of 2.053 nm and 2.044 nm, respectively (Figure 8B). These findings suggest that the isolate complexed with β-ketoacyl-ACP synthase from *Escherichia coli* maintained a constant Rg value while exhibiting higher compactness compared to TyrRS from *Streptococcus aureus*.

##### Protein–Ligand Interaction

**Figure 9:**
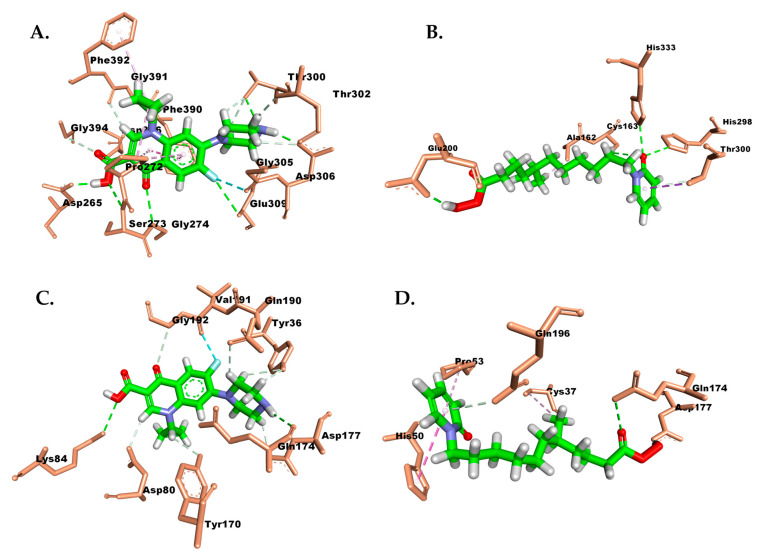
Molecular interactions between protein–ligand complex of (**A**) ciprofloxacin and (**B**) isolate in β-ketoacyl-ACP synthase from *Escherichia coli* and between (**C**) ciprofloxacin and (**D**) isolate in TyrRS from *Streptococcus aureus*. Carbon, oxygen, and nitrogen atoms are marked in green, red, and blue. Amino acid residues are highlighted with a light brown color. The green, gray, and pink dotted lines represent hydrogen bonding, carbon-hydrogen bonds, and hydrophobic interactions, respectively.

To obtain protein–ligand interactions during molecular dynamics, trajectory extraction was performed at the end of the dynamic simulation. Figure 9 represents the interaction fractions between the isolate and β-ketoacyl-ACP synthase from *Escherichia coli* and TyrRS from *Streptococcus aureus*, as observed in the dynamics simulation results.

The interaction of ciprofloxacin with β-ketoacyl-ACP synthase from *Escherichia coli* revealed the formation of new hydrogen bonds with residues Asn396, Asp265, Ser273, Gly274, Thr300, Asp306, and Gly391, while maintaining the hydrogen bonds with residues Gly394 and Glu309, as observed in the docking results. Meanwhile, in the case of the isolated compound, there were changes in hydrophobic interactions, which transformed into hydrogen bonds with residues Cys163 and His333. Additionally, there was an increase in hydrogen bond formation and enhanced hydrophobic interactions with residues Glu200, His298, Ala162, and Thr300, which were not observed in the docking results.

The molecular dynamics results demonstrate an enhanced formation of hydrogen bonds by analyzing the interactions based on the molecular dynamics simulation results of the ciprofloxacin in TyrRS from *Streptococcus aureus*. The bonds with residues Asp177 and Gly193 were maintained while exhibiting additional interactions with residues Gly174, Lys84, Gln190, Tyr170, and Gly192 (Figure 9C). Conversely, the isolated compound reveals the emergence of novel hydrogen bonds with residues Asn124, Asp177, and Gln174, which were not observed in the docking results. Additionally, the hydrophobic interactions with residues Pro53 and Cys37 remain preserved (Figure 9D).

This study clearly demonstrates significant changes in the interactions of the isolated compound with *Escherichia coli* and *Streptococcus aureus* targets, providing deeper insights into the molecular mechanisms of the isolated compound obtained from *S. trifasciata.*

##### Binding Free-Energy Enzyme Compounds

The binding energy of the isolated compound was calculated using the MM-PBSA method to evaluate the contributions of each energy component to the overall change in free energy (Table 2). This analysis helps in understanding how the interactions between the protein and the ligand contribute to the total binding energy of the system. The total binding energy (∆E_Bind_) of the isolated compound in the β-ketoacyl-ACP synthase target from *Escherichia coli* was better than that of the native ligand (TLM) but slightly weaker than that of the ciprofloxacin. On the other hand, in the TyrRS system from *Streptococcus aureus*, the isolate exhibited weaker affinity compared to the native ligand (629) and ciprofloxacin. Interestingly, although weak, the isolated compound showed a total free energy that was nearly equivalent to ciprofloxacin in both the *Escherichia coli* and *Streptococcus aureus* enzymes. In the β-ketoacyl-ACP synthase system from *Escherichia coli*, the isolate had a total free energy of −104.101 kJ/mol, while in the TyrRS system from *Streptococcus aureus*, it was −81.060 kJ/mol. The van der Waals energy (∆E_VDW_), electrostatic energy (∆E_Ele_), and SASA energy (∆E_SASA_) had positive impacts on the total free energy of both the β-ketoacyl-ACP synthase and TyrRS systems. Meanwhile, the polar solvation energy (∆E_PB_) contributed to a more positive total binding energy of the system.

### 2.4. Antibacterial Activity

**Figure 10:**
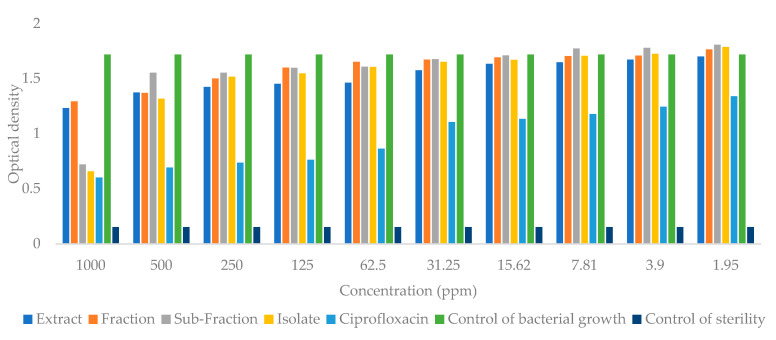
Graph of optical density of *Escherichia coli* bacteria after being given extracts, fractions, subfractions, isolates, and ciprofloxacin.

**Figure 11:**
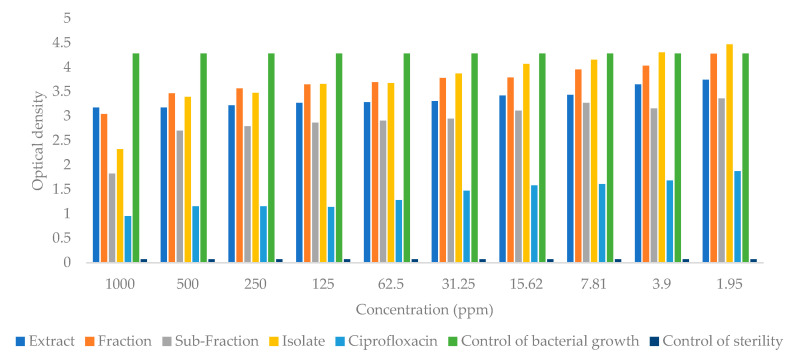
Graph of optical density of Streptococcus aureus bacteria after being given extracts, fractions, subfractions, isolates, and ciprofloxacin.

The extracts, fractions, subfractions, and isolates obtained from the leaves of *S. trifasciata* Prain were used in the antibacterial testing of *Streptococcus aureus* and *Escherichia coli* bacteria with the microdilution method. The level of turbidity (optical density) was measured at 24 h with a standard of 0.5 McFarland. The value of the optical density is proportional to the growth of bacteria. Based on the graph of the growth of *Escherichia coli* bacteria (Figure 10) the concentration can reduce the growth of bacteria by decreasing the value of optical density. The growth of *Escherichia coli* bacteria decreased significantly (*p* < 0.05), with the lowest growth seen in the group given the isolate, followed by subfractions, extracts, and fractions, respectively. The results of the optical density of *Streptococcus aureus* bacteria (Figure 11) show that the bacterial growth decreased in the group given the subfraction, followed by isolates, fractions, and extracts, respectively.

The results of determining the minimum inhibitory concentration (MIC) value for *Escherichia coli* bacteria showed that the extracts, fractions, subfractions, and isolates had MIC values of 1.95 ppm each; 3.9 ppm; 15.62 ppm; 7.81 ppm respectively. The MIC values of the *Streptococcus aureus* bacteria for each sample were 1.95 ppm, 1.95 ppm, 15.62 ppm, and 7.81 ppm, respectively. The minimum bactericidal concentration (MBC) value of the sample was not obtained because the absorbance obtained from the test was not close to zero.

## 3. Discussion

*S. trifasciata* Prain is an important plant with many bioactive compounds that have therapeutic properties. There have been reports of pharmacological activities, including anti-alopecia via the inhibition of an androgen receptor [27], a hepatoprotector, by activating the NRF2/ARE signaling pathway [28]. Further identification of the chemical compound responsible for anti-alopecia activity has occurred. Infrared spectroscopy analysis revealed the presence of steroid and terpenoid compounds with anti-alopecia properties [29]. In this study, the bioactive compound isolated from the ethyl acetate fraction was an alkaloid derivative, namely 5-methyl-11-(2-oxopyridine-1(2H)-yl)undecaneperoxoic acid. Intriguingly, neither the discovery nor the pharmacological activity of this compound has previously been reported. Therefore, it is imperative to explore its potential.

Compound 5-methyl-11-(2-oxopyridine-1(2H)-yl)undecaneperoxoic acid is a novel chemical entity that has not been previously reported in any known plant species. This research focuses on unraveling the structure of this compound, and during the study, a significant finding emerged, namely the presence of a chiral atom on the C-5 atom. This specific carbon atom is crucial as it is the central stereogenic chiral carbon with four distinct substituents. The researchers established a priority order for the substituents to understand the compound's structure better based on their respective molecular weights. Notably, the substituent containing oxo pyridine exhibited the highest molecular weight, granting it the utmost significance and making it the primary priority. Following precedence are peroxidic acid, a methyl group, and the hydrogen atom, which holds the lowest priority. The analysis of the stereochemistry of this compound hinges on the sequence determined by the molecular weights of the substituents. Consequently, the most likely conformation for this compound is the (*S*) conformation, which is attributed to the arrangement dictated by the order of molecular weights [30].

This study examined the antibacterial activity of extracts, fractions, subfractions, and isolates against *Escherichia coli* and *Streptococcus aureus*. According to the results, the fractions inhibited growth less than the other samples, and this may be due to the antagonistic effect of the compounds in the fraction, which can reduce the pharmacological effect [31]. In order to maximize the pharmacological effect, the sample fraction was further separated into subfractions. Comparing the results of the subfraction group to the results of the fraction group (Figure 10 and Figure 11), bacterial proliferation decreased in the subfraction group.

Isolates containing 5-methyl-11-(2-oxpyridine-1(2H)-yl)undecaneperoxoic acid exhibited significant inhibitory activity. As shown in Figure 2, the isolate had the lowest bacterial growth together with ciprofloxacin. This indicates that this compound is promising for further development as an *Escherichia coli* antibacterial treatment. The structure of this compound contains 2-pyridone alkaloid, which is known to possess antibacterial activity. In a different study, a 2-pyridone derivative compound inhibited the growth of *Streptococcus aureus* bacteria [32], and it inhibited the growth of *Escherichia coli* on the DNA gyrase enzyme better than tetracycline based on the results of an in silico study [33]. In addition, as an antibacterial, 4-hydroxy-2-pyridone, a derivative of 2-pyridone, has shown antitumor [34] and anticancer [35] activity.

Molecularly, the isolate is capable of occupying the active site of β-ketoacyl-ACP synthase from *Escherichia coli* and that of TyrRS from *Streptococcus aureus*. This is evidenced by its interactions with the active site residues of β-ketoacyl-ACP synthase, such as His333, Cys163, and Phe392. Additionally, the terpenoid tail of the isolated compound can also interact with Pro272 residue, resembling the interaction observed with TLM. Meanwhile, in TyrRS, the isolated compound can mimic the interactions of the TyrRS inhibitor (629) by forming hydrogen bonds with Asp40, Asp80, and Gly193, as well as hydrophobic interactions with Leu70.

The results of the molecular dynamics simulations further support the previously predicted affinity, where the isolated compound exhibited excellent stability when interacting with β-ketoacyl-ACP synthase from *Escherichia coli* and with TyrRS from *Streptococcus aureus* (Figure 5). However, it is also noted that the isolated compound induced increased fluctuations in certain residues, such as Thr57 and Lys40 in β-ketoacyl-ACP synthase, and residues Arg88, His117, Glu160, and Gln213 in TyrRS. The molecular interaction profile of the isolated compound with β-ketoacyl-ACP synthase from *Escherichia coli* revealed fascinating discoveries. The transformation of hydrophobic interactions into hydrogen bonds with residues Cys163 and His333 indicates a shift in the binding mode. Additionally, the molecular dynamics simulation demonstrated an increased formation of hydrogen bonds and intensified hydrophobic interactions with residues Glu200, His298, Ala162, and Thr300 (Figure 9B). These findings emphasize the dynamic nature of the binding process and the potential for conformational changes. Furthermore, the interaction of the isolated compound with TyrRS from *Streptococcus aureus* exhibited the formation of intriguing hydrogen bonds with residues Asn124, Asp177, and Gln174. This observation highlights the dynamic nature of the system, as these newly formed hydrogen bonds could significantly impact the compound’s binding affinity and stability. Moreover, the crucial hydrophobic interactions with residues Pro53 and Cys37 remained consistently maintained throughout the simulation (Figure 9D), suggesting their vital role in stabilizing the complex and preserving its overall structural integrity. The computational study provided information consistent with the in vitro assays, where the binding energy calculated using the MMPBSA approach indicates that the isolated compound had affinity levels comparable to ciprofloxacin (Table 2). Based on these findings, it is estimated that the isolated compound possesses potential antibacterial activity by inhibiting the enzymatic activities of β-ketoacyl-ACP synthase from *Escherichia coli* and those of TyrRS from *Streptococcus aureus*. To assess the efficiency of this isolated compound, a combination of in silico and in vitro studies is needed, especially for many other types of bacteria, to reveal more of its potential as an antibacterial agent.

## 4. Materials and Methods

### 4.1. Maceration and Isolation

The leaves of *S. trifasciata* Prain were drained, weighed, and ground into dry simplicia powder. The simplicia was macerated for 36 h in 95% ethanol (*v*/*v*), then filtered and concentrated using a rotary evaporator (Buchi^®^, Flawil, Switzerland) into a crude extract, and the yield was calculated. The ethanol extract of *S. trifasciata* Prain was fractionated by the trituration method. The crude extract (50 g) was put into a mortar, and n-hexane was added while grinding the extract with a pestle. Then, the filtrate and residue were separated, where the filtrate was collected as the n-hexane fraction, and the residue was crushed together with ethyl acetate solvent. The ethyl acetate filtrate was concentrated using a rotary evaporator to obtain a crude fraction. The ethyl acetate fraction was then separated by the vacuum column chromatography technique using silica gel 60 GF254 (Merck^®^, Darmstadt, Germany) and eluted with a mixture of n-hexane/ethyl acetate (0:100%, 10:90%, 20:80%, 30:70%, 40:60%, 50:50%, 40:60%, 30:70%, 20:80%, 10:90%, 0:100%) and ethyl acetate/methanol (0:100%, 10:90%, 20:80%, 30:70%, 40:60%, 50:50%, 40:60%, 30:70%, 20:80%, 10:90%, 0:100%). The results of these separations yielded nine (A-I) combined subfractions. Subfraction F (1.91 g) was further separated by radial chromatography (Kromatron^®^, Annonay, France), eluted with n-hexane/ethyl acetate (3:7), and five subfractions (F1-F5) were obtained. The subfractions F1, F3, and F4 were combined to form FA, while F2 and F5 were combined to become FB. The FA subfraction was used for further separation. The results of the FA separation obtained 21 subfractions. Subfractions 12–21 were combined to become FA1 and further separated to produce 12 subfractions (Fa1-Fal2), and subfractions 6–9 were combined as an isolate with a single spot (12.6 mg). The separation profiles of the isolate were visualized in TLC with a UV lamp (UVG-58).

### 4.2. Isolate Characterization

The isolate characterization was carried out based on the results of the 1D and 2D H-NMR and the C-NMR spectra (JOEL JNM-ECZ500R/S1), namely by distortionless enhancement by polarization transfer-135 (DEPT), heteronuclear multiple quantum coherence (HMQC), heteronuclear multiple bound correlation (HMBC), and ^1^H-^1^H correlation spectroscopy (^1^H-^1^H COSY).

#### 5-Methyl-11-(2-oxopyridin-1(2H)-yl)undecaneperoxoicacid: Yellow Oil Form

^1^H-NMR (DMSO, 500 MHz), δH 7.74 (1H, d, J = 8, H-3′), 7.03 (1H, d, H-6′), 6.94 (1H,d, H-5′), 6.77 (1H, d, J = 8, H-4′), 2.24 (1H, s, H-2), 1.47 (2H, s, H-3), 1.27 (1H, d, J = 9.5, H-5), 1.19 (1H, H-6), 1.19 (1H, H-7), 1.19 (1H, H-8), 1.19 (1H, H-9), 1.19 (1H, H-10), 1.19 (1H, H-11), 0.79 (3H, 5-CH3), 3.94 (1H, s, -OH).

^13^C-NMR (DMSO, 125 MHz): δC 173.2 (C-1), 162.084 (C-2′), 132.069 (C-3′), 126.972 (C-5), 125.955 (C-6′), 115.646 (C-4′), 33.731 (C-2′), 31.83 (C-11), 29.872 (C-5), 29.546 (C-6), 29.440 (C-10), 29.277 (C-8), 29.248 (C-9), 29.085 (C-7), 24.458 (C-3), 22.635 (C-4), 14.495 (C-12).

### 4.3. Computational Study

#### 4.3.1. Molecular Docking Study

We selected the crystallographic structures of β-ketoacyl-acyl carrier protein synthase (β-ketoacyl-ACP synthase) and tyrosyl-tRNA synthetase (TyrRS) with Protein Data Bank IDs 1FJ4 and 1JIJ [36,37]. Water molecules and bound ligands were removed from the enzymes. In AutoDock Tools v1.5.6, the target protein was protonated by adding polar hydrogen atoms and Kollman charges [38]. Meanwhile, the structures of the isolated compound and ciprofloxacin (ligand) were drawn using ChemDraw and optimized in HyperChem with AM1 methods. Lastly, hydrogen atoms were added to the ligand structures, and the Gasteiger charges were adjusted using AutoDock Tools v1.5.6 [39].

The binding affinity and interactions of the isolated compounds in *S. trifasciata* were determined using AutoDock. The native ligands (TLM and 629) were redocked to the β-ketoacyl-ACP synthase and TyrRS, respectively, to validate the docking parameters. The validated procedures were identified with a root mean square deviation (RMSD) below 2 Å [40]. The binding site of β-ketoacyl-ACP synthase and TyrRS were set according to their native ligand positions and calculated using a cubic shape with a grid area of 40 × 40 × 40 Å. The proteins’ and ligands’ hydrogen bonding and hydrophobic interactions were analyzed and visualized employing Discovery Studio Visualizer v.17.2.0.16349 software.

#### 4.3.2. Molecular Dynamics Simulation

GROMACS 2021.3 software was utilized to perform molecular dynamics simulations of the β-ketoacyl-ACP synthase and TyrRS systems [41]. The protein topology was generated using the AMBER99SB-ILDN force field [42], while the ligand topology was generated using the General Amber force field [43] with the assistance of Antechamber in AmberTool 2021, which was executed in ACPYPE [44]. The solvation process employed the TIP3P water model at a temperature of 310K and was neutralized by the addition of Na and Cl ions [45]. An electrostatic force was simulated using the Particle Mesh Ewald method [46]. The complex’s stability was assessed by analyzing parameters such as the root mean square deviation (RMSD), root mean square fluctuation (RMSF), radius of gyration (Rg), solvent accessible surface area (SASA), and principal component analysis (PCA) of the trajectory during a 100 ns simulation. Lastly, the binding energies of the protein–ligand system were determined using the MM/PBSA methodology through the g_mmpbsa packages [47].

### 4.4. Antibacterial Activity

An antibacterial activity assay was carried out using the microdilution method with slight modification [48]. The extract, ethyl acetate fraction, subfraction G, and isolate from *S. trifasciata* Prain leaves were used as samples, with ciprofloxacin as a positive control. *Streptococcus aureus* and *Escherichia coli* bacteria were obtained from the Biomedical Laboratory of the Faculty of Medicine, Universitas Halu Oleo. Mueller Hinton Broth (MHB) medium (50 µL) was first put in a microplate (Greiner Bio-One^®^, Kremsmünster, Austria), and then each sample (50 µL) was added with various concentrations of 1000 ppm, 500 ppm, 250 ppm, 125 ppm, 62.5 ppm, 31.25 ppm, 15.62 ppm, 7.81 ppm, 3.8 ppm, and 1.95 ppm. The bacterial suspension (100 µL), positive control (100 µL) and media growth control (100 µL) were then added to the well plate. The microplates were incubated (37 °C for 24 h), and the absorbance or optical density was measured at 625 nm using a spectrophotometer ELISA reader (Thermo Scientific^®^, Waltham, MA, USA). The determination of the minimum inhibitory concentration (MIC) and minimum bactericidal concentration (MBC) was carried out by calculating the turbidity based on the absorbance value.

### 4.5. Data Analysis

The antibacterial activity data in the form of the absorbance/optical density of *Escherichia coli* and *Streptococcus aureus* bacteria were analyzed using the IBM^®^ (Armonk, NY, USA) Statistics SPSS application version 24.

## 5. Conclusions

Extracts, fractions, subfractions, and isolates can inhibit the growth of *Escherichia coli* and *Streptococcus aureus* bacteria. Compound 5-methyl-11-(2-oxpyridine-1(2H)-yl)undecaneperoxoic acid can be developed as an antibacterial against *Escherichia coli* and *Streptococcus aureus*. According to the results, the isolate had a high affinity for β-ketoacyl-ACP synthase from *Escherichia coli* and for TyrRS from *Streptococcus aureus*, as evidenced by its strong binding and interactions with key amino acid residues. Furthermore, stable complexes were generated during dynamic modeling, which serves as a springboard for further investigation into the compound’s content and activity in *S. trifasciata* plants, opening up new avenues for research in the development of novel antibacterial drugs.

## Figures and Tables

**Table 1:** Summary of docking results from isolate to the crucial enzyme of *Escherichia coli* and *Streptococcus aureus*.

β-Ketoacyl-ACP Synthase from *Escherichia coli*		TyrRS from *Streptococcus aureus*	
Compounds	Binding Energy (kcal/mol)	Compounds	Binding Energy (kcal/mol)
TLM	−7.96	629	−9.35
Ciprofloxacin	−6.81	Ciprofloxacin	−6.69
Isolate	−6.72	Isolate	−6.15

**Table 2:** The binding energies of all complex systems during 100 ns dynamic simulation.

β-Ketoacyl-ACP Synthase from *Escherichia coli* (All Energies Are in kJ/mol)					
Compounds	van der Waal	Electrostatic	Polar Solvation	SASA	Binding Energy
TLM	−116.620	−41.128	86.225	−13.079	−84.603
Ciprofloxacin	−179.729	−102.199	192.537	−16.739	−106.131
Isolate	−153.888	−46.728	114.871	−18.356	−104.101
**TyrRS from *Streptococcus aureus* (All Energies Are in kJ/mol)**					
**Compounds**	**van der Waal**	**Electrostatic**	**Polar Solvation**	**SASA**	**Binding Energy**
629	−207.705	−116.709	257.610	−19.976	−86.780
Ciprofloxacin	−205.931	−86.473	228.331	−18.094	−82.167
Isolate	−193.642	−49.507	182.081	−19.992	−81.060

## Data Availability

Data are contained within the article and Supplementary Materials.

## Supplementary Materials

The following supporting information can be downloaded at: https://www.mdpi.com/article/10.3390/molecules28166096/s1, Figure S1: H-NMR spectra of 5-methyl-11-(2-oxopyridine-1(2H)-yl)undecaneperoxoicacid, Figure S2: C-NMR spectra of 5-methyl-11-(2-oxopyridine-1(2H)-yl)undecaneperoxoicacid, Figure S3: DEPT 135 spectra of 5-methyl-11-(2-oxopyridine-1(2H)-yl)undecaneperoxoicacid, Figure S4: HMQC spectra of 5-methyl-11-(2-oxopyridine-1(2H)-yl)undecaneperoxoicacid, Figure S5: HMBC spectra of 5-methyl-11-(2-oxopyridine-1(2H)-yl)undecaneperoxoicacid, Figure S6: COSY spectra of 5-methyl-11-(2-oxopyridine-1(2H)-yl)undecaneperoxoicacid.
