# Supplementary material for: Antibacterial Potency of an Active Compound from Sansevieria trifasciata Prain: An Integrated In Vitro and In Silico Study
Source: Molecules. 2023 Aug 17;28(16):6096. doi: 10.3390/molecules28166096 (PMC10457997; doi:10.3390/molecules28166096)
Supplement: Supplementary file 1 [file molecules-28-06096-s001.zip › molecules-2505834-supplementary.pdf]

molecules

MDPI

Supplementary Materials

Antibacterial Potency of an Active Compound from *Sansevieria trifasciata* Prain: An Integrated In Vitro and In Silico Study

Henny Kasmawati [1,*], Ruslin Ruslin [1], Arfan Arfan [1], Nurramadhani A. Sida [1], Dimas Isnu Saputra [1], Eli Halimah [2] and Resmi Mustarichie [3,*]

[1] Department of Pharmacy, Faculty of Pharmacy, Universitas Halu Oleo, Kendari 93232, Indonesia; mahaleo241@yahoo.co.id (R.R.); arfan09@uho.ac.id (A.A.); apt.nurramadhani08@uho.ac.id (N.A.S.); dimasisnusaputra@gmail.com (D.I.S.)

[2] Department of Pharmacology and Clinical Pharmacy, Faculty of Pharmacy, Universitas Padjadjaran, Bandung 45363, Indonesia; eli.halimah@unpad.ac.id

[3] Department of Analytical Pharmacy and Medicinal Chemistry, Faculty of Pharmacy, Universitas Padjadjaran, Bandung 45363, Indonesia

* Correspondence: hennykasmawati@uho.ac.id (H.K.); resmi.mustarichie@unpad.ac.id (R.M.)

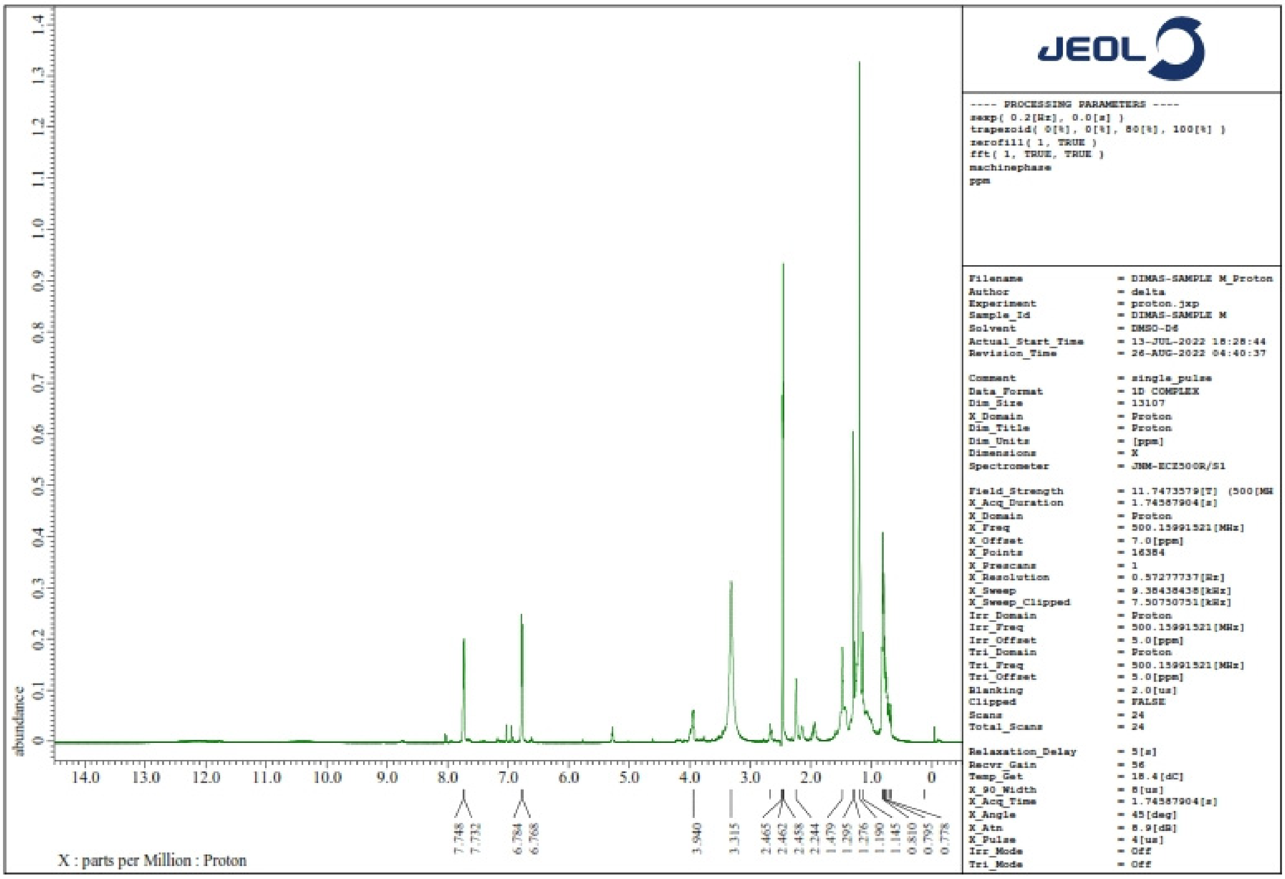

Figure S1. H-NMR spectra of 5-methyl-11-(2-oxopyridine-1(2H)-yl)undecaneperoxoicacid.

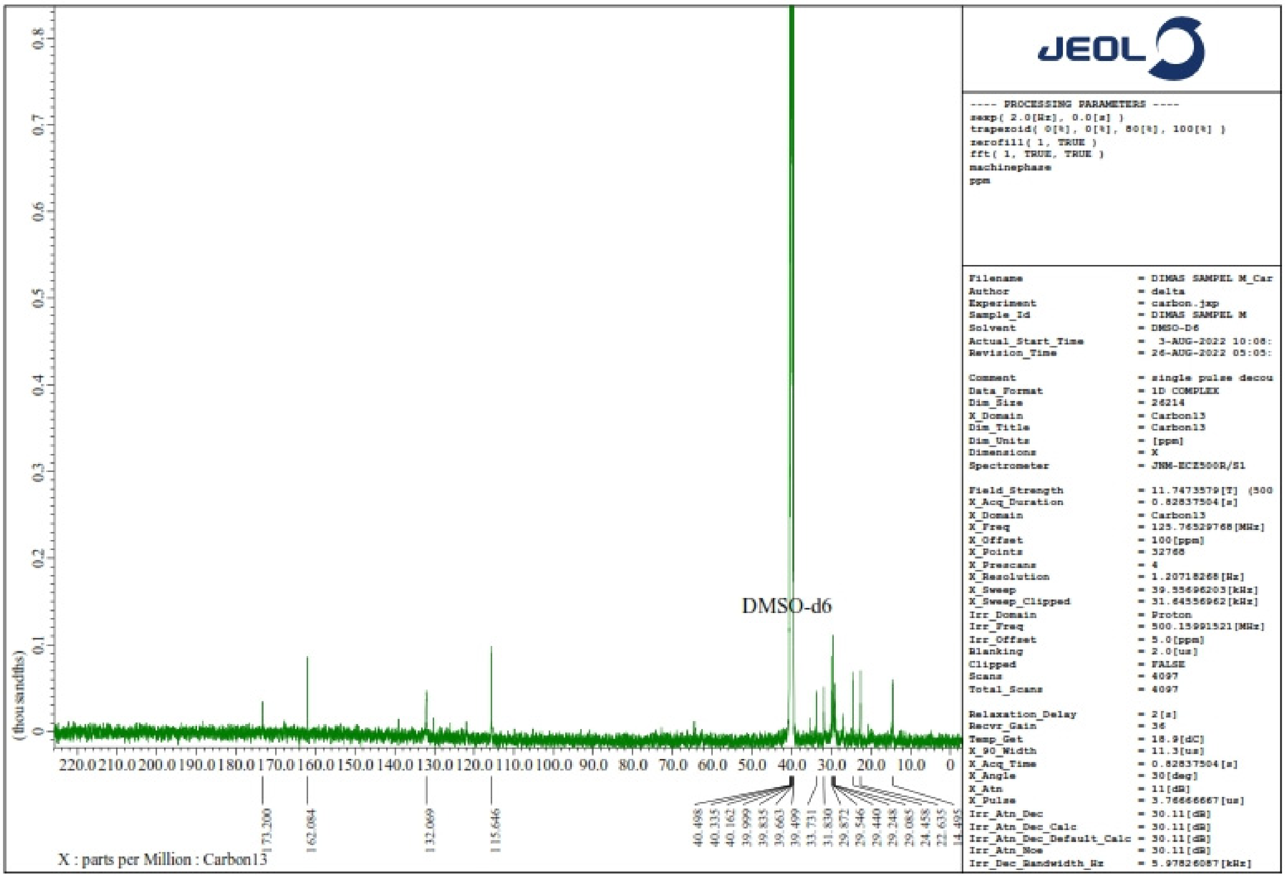

Figure S2. C-NMR spectra of 5-methyl-11-(2-oxopyridine-1(2H)-yl)undecaneperoxoicacid.

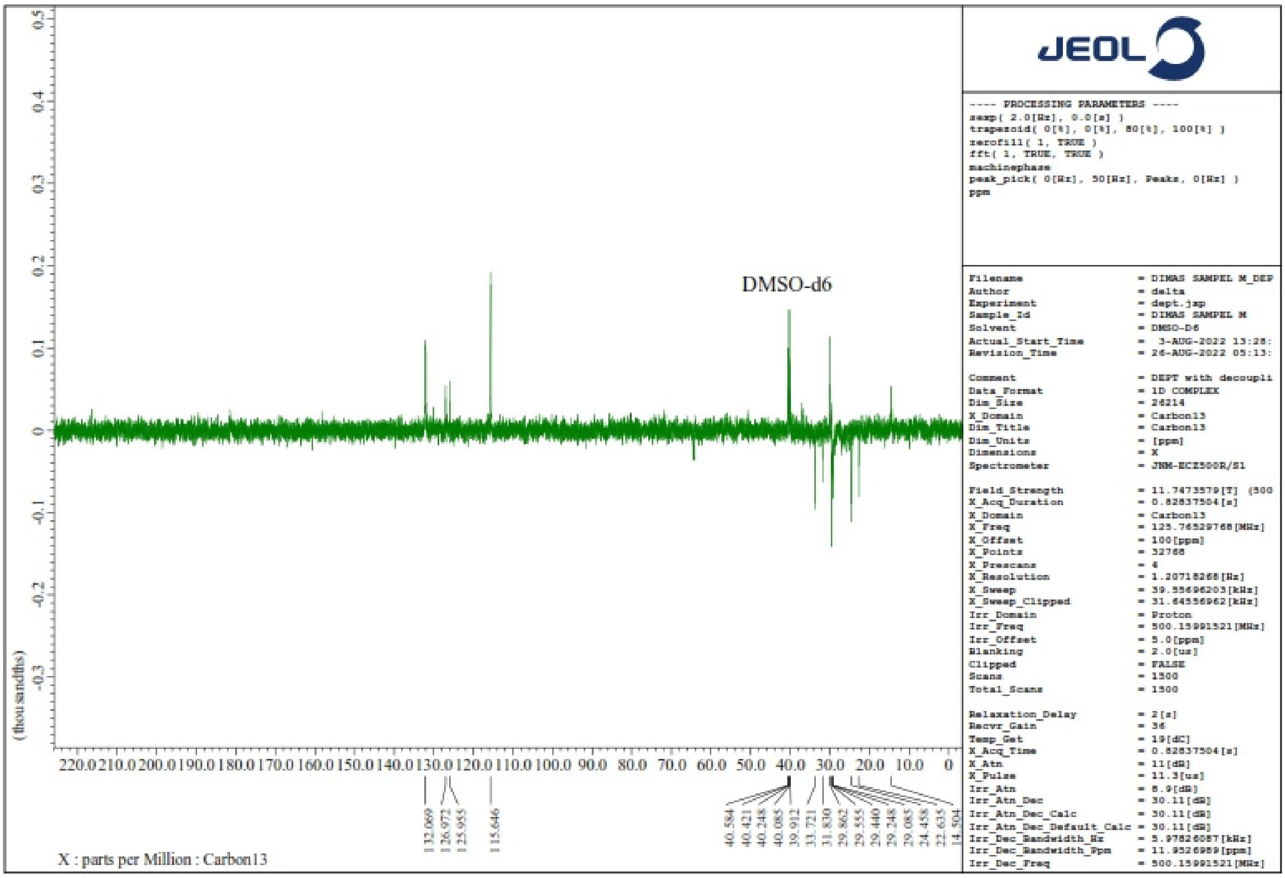

Figure S3. DEPT 135 spectra of 5-methyl-11-(2-oxopyridine-1(2H)-yl)undecaneperoxoicacid.

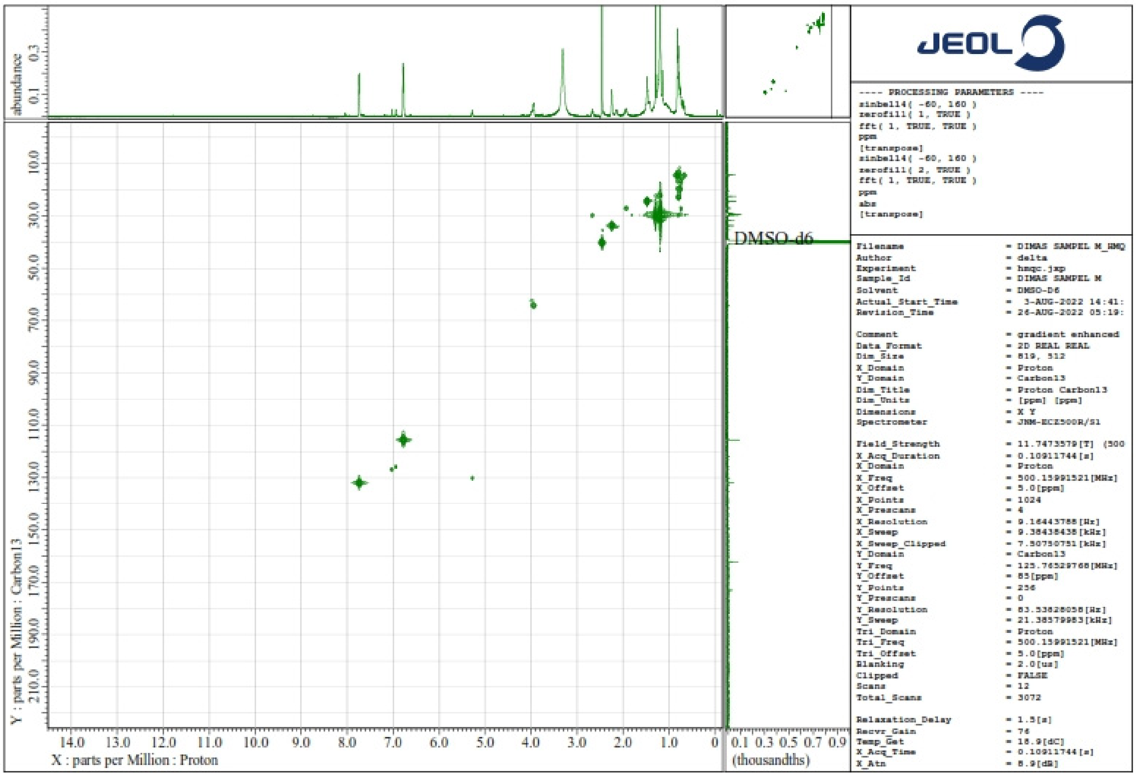

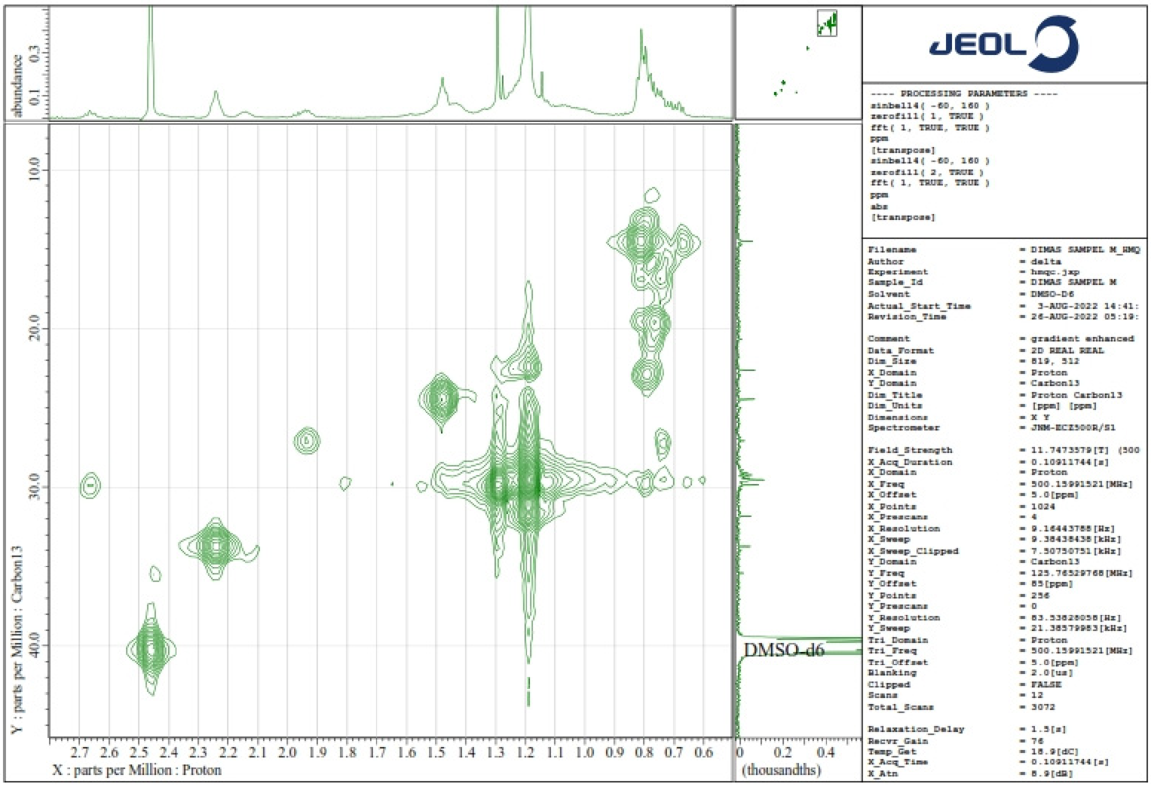

Figure S4. HMQC spectra of 5-methyl-11-(2-oxopyridine-1(2H)-yl)undecaneperoxoicacid.

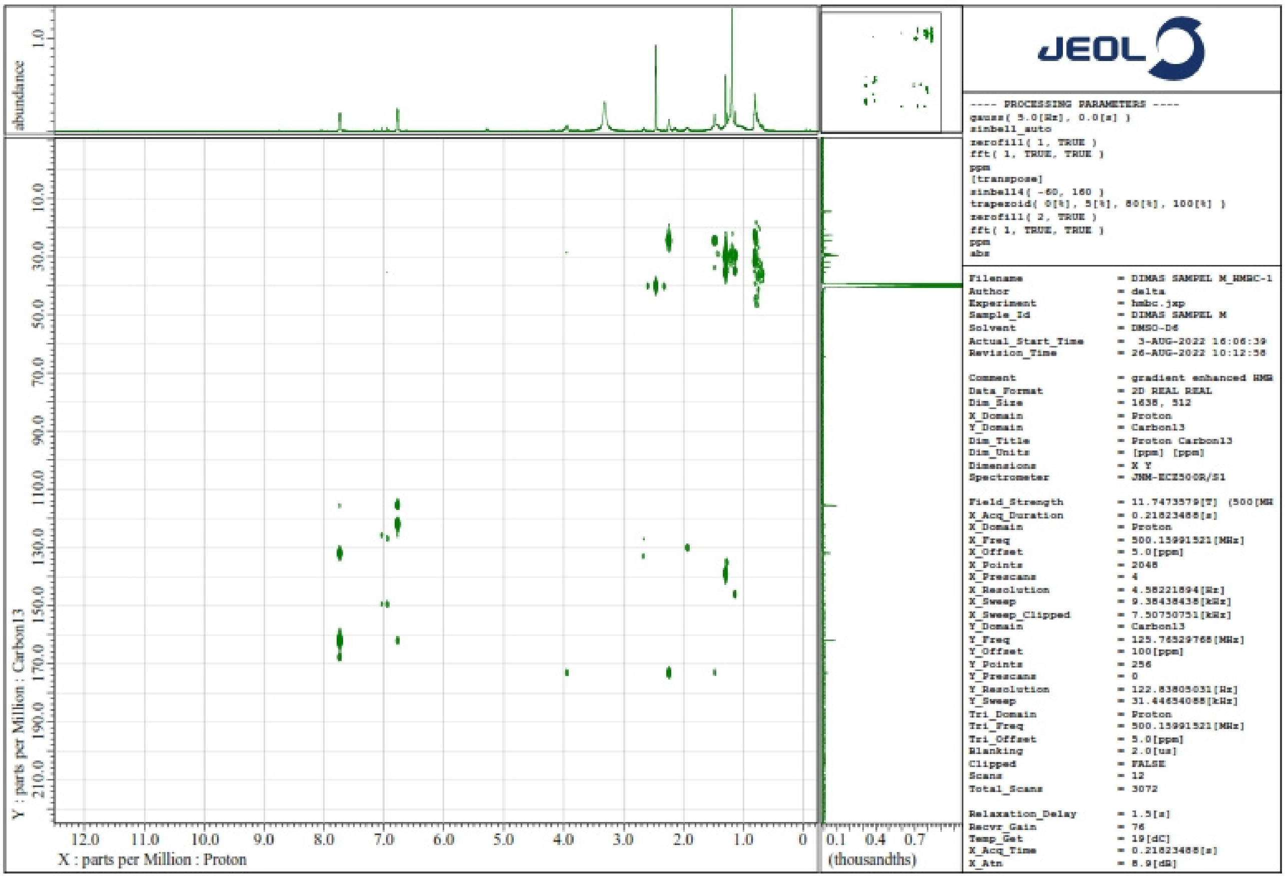

Figure S5. HMBC spectra of 5-methyl-11-(2-oxopyridine-1(2H)-yl)undecaneperoxoicacid.

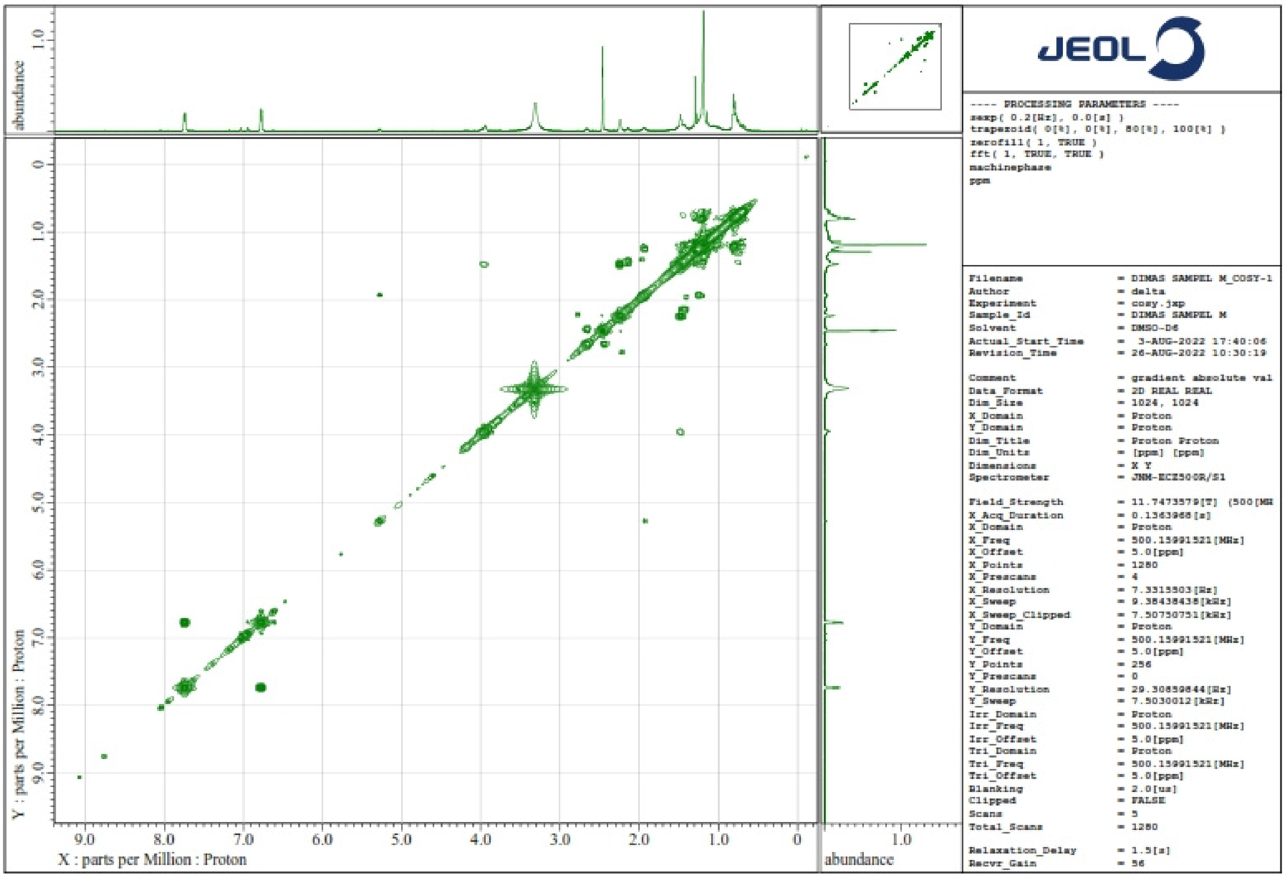

Figure S6. COSY spectra of 5-methyl-11-(2-oxopyridine-1(2H)-yl)undecaneperoxoicacid.
